# Orbital Decompression in Thyroid Eye Disease

**DOI:** 10.5402/2012/739236

**Published:** 2012-11-12

**Authors:** N. Fichter, R. F. Guthoff, M. P. Schittkowski

**Affiliations:** ^1^Interdisciplinary Center for Graves' Orbitopathy, Admedico Augenzentrum, Fährweg 10, 4600 Olten, Switzerland; ^2^Department of Ophthalmology, University of Rostock, Doberaner Strasse 140, 18055 Rostock, Germany; ^3^Department of Strabism, Neuro-Ophthalmology and Oculoplastic Surgery, University of Goettingen, Robert-Koch-Stra*β*e 40, 37075 Göttingen, Germany

## Abstract

Though enlargement of the bony orbit by orbital decompression surgery has been known for about a century, surgical techniques vary all around the world mostly depending on the patient's clinical presentation but also on the institutional habits or the surgeon's skills. Ideally every surgical intervention should be tailored to the patient's specific needs. Therefore the aim of this paper is to review outcomes, hints, trends, and perspectives in orbital decompression surgery in thyroid eye disease regarding different surgical techniques.

## 1. Introduction

Thyroid-associated orbitopathy (TAO) can be functionally disabling and, in severe cases, may result in permanent visual loss. It may also cause significant facial disfigurement.

Although many authors use the term “*cosmetic decompression*,” it must be kept in mind that this in fact represents *reconstructive surgery* because it addresses an abnormality caused by a disease. While decompression surgery has the potential to improve facial appearance, patients should be carefully informed that it is often impossible to restore their look to what it has been before the disease began to modify the tissues involved. Frequently multiple surgical procedures are required, ranging from orbital decompression surgery via strabismus surgery to lid surgery, again emphasizing that these procedures are not performed for the purpose of “*beautification*” [1].

It is important to note that most TAO patients will not require surgical treatment. In 1996 Bartley et al. [2] demonstrated that only 20% of their patients had one or more surgical procedures. The cumulative probability of having surgery was initially 5% by 1 year after first diagnosis of the disease, rising to 9.3% by 2 years, to 15.9% by 5 years, and to 21.8% by 10 years. The need for surgery was significantly related to age, with a 2.6 times greater overall risk in patients older than 50 years.

Medical (immunosuppressive) measures are the first-line treatment in the active stage of TAO. In the in-active stage of the disease or if medical therapy fails in sight threatening situations in active eye disease, the surgeon will be called upon to improve the patient's condition. 

This paper aims to review historical aspects and recent advances in decompression surgery in thyroid associated orbitopathy.

## 2. Clinical Findings and Indications for Orbital Decompression

Treatment of TAO requires an accurate assessment of disease activity, temporal progression, and severity. The aim of diagnosis is to differentiate the active stage—which represents a potential threatening of visual functions—from the inactive “burnt-out” stage of the disease. 

Active moderate or severe congestive orbitopathy usually asks for immediate intervention, whereas active mild orbitopathy may only require supportive measures and a period of observation to discover whether disease is improving or worsening [3, 4]. 

Sight-threatening dysthyroid optic neuropathy (DON) occurs in about 5% of patients with Graves' disease. Clinical findings can be loss of visual acuity or colour vision deficiency, visual field defects, relative afferent pupillary defect, or optic disc swelling. DON can be confirmed by visual evoked potentials with a significant increase in latency and/or reduction of amplitude. Without treatment, irreversible visual loss occurs in 30% of these cases [5]. Older age, male gender, and smoking are important factors associated with an increased risk for DON [2, 6]. 

The most widely accepted pathophysiologic mechanism for optic nerve involvement is compression of the nerve or its blood supply by the orbital contents in the orbital apex, mainly the extraocular muscles (EOMS). Many studies have shown a relationship between muscle size, restriction of motility, and DON, while proptosis itself did not correlate well with the risk for DON. 

Because of the potential risk for blinding DON requires immediate intervention. Wakelkamp et al. [7] demonstrated in a randomized clinical trial that in the event of DON immediate decompressive surgery does not result in a better outcome compared to medical immunosuppressive treatment. Therefore high-dose intravenous methylprednisolone therapy is recommended as the first-line treatment. However, if medical treatment does not improve visual functions within a few days or if there is a further deterioration, surgery appears to be the only way to avoid persistent visual loss due to optic nerve atrophy. The apical syndrome with congestion of the optic nerve in the orbital apex is best treated by a mechanical decompression that addresses the location of the compressive component, that is, by resection of the medial wall in the deep orbit. In those rare instances where DON occurs in the absence of apical compression, increased orbital pressure may be a causative factor in the sense of an orbital compartment syndrome (see the following). Appropriate imaging techniques, for example, MRI, are mandatory for differentiating DON caused by apical compression or by compartment syndrome. 

In the absence of DON elective reconstructive surgery for exophthalmos reduction or to relieve diffuse retrobulbar pressure sensation is usually considered after ophthalmological findings have been stable for at least 3–6 months. Early rehabilitative orbital decompression does not improve surgical outcome and is associated with a higher risk of induced motility problems [8]. In general, if orbital decompression is needed, it has to be performed before EOM or eyelid surgery because it can affect both extraocular muscle balance and eyelid position [9].

## 3. Pathophysiological Aspects of the Orbital Compartment Syndrome

The orbit is an enclosed cone-shaped compartment bounded by bone posterocircumferentially and by the orbital septum anteriorly. The latter tight structure allows only limited forward displacement of the eye in response to increased orbital volume, such as what occurs in TAO. The intact orbital septum can withstand experimental pressures of 50 mm Hg and up to 120 mm Hg in some cases [10]. By analogy with the pathophysiological processes described in a surgical and orthopaedic setting that terms an increased tissue pressure in an enclosed space as an “compartment syndrome,” Kratky et al. [11] first transferred this term to certain orbital conditions and described it as the “orbital compartment syndrome” in 1990: “Because of their confined anatomy, the orbital contents display the pressure-volume dynamics of a closed compartment. In some cases a significant rise in intraorbital pressure may compromise the perfusion of susceptible tissues and result in visual loss.” Orbital pressure is measured to be 3–6 mm Hg in healthy individuals and at 7–15 mm Hg in TAO patients [11]. The final common pathway to visual loss in orbital compartment syndrome appears to be damage to the optic nerve fibres. Inadequate blood flow in the posterior ciliary arteries, the central retinal artery or vein, or the vasa nervorum of the optic nerve results in a variety of clinical presentations, including ischaemic optic neuropathy, central retinal artery or vein occlusion, or slow cavernous optic nerve degeneration.

Riemann et al. [12] determined orbital tissue pressure of 4.0 ± 1.5  mm Hg in a larger series of 18 healthy orbits. Orbital compliance was 0.74 ± 0.31 mL/mm Hg after retrobulbar injection of local anaesthetic. In a later study the same authors showed that resting orbital tissue pressure was 9.7 ± 4.8 mm Hg in TAO patients and, of greater practical importance as a reaction to a further increase in volume, orbital compliance was significantly lower with 0.27 ± 0.21 mL/mm Hg [13]. Resting orbital tissue pressure was even higher in TAO patients with DON (12.4 ± 4.9 mm Hg), thus demonstrating the validity of this concept.

DON may result partially—or in certain selected cases totally—from an orbital compartment syndrome, and it should be kept in mind that orbital imaging without signs of apical EOM crowding and consecutive optic nerve congestion does not exclude DON. DON is a clinical diagnosis related to a disturbance of visual functions as reduction in visual acuity and/or colour vision, visual field defects, relative afferent pupil defects, and disc congestion. Patients with apical muscle crowding might benefit from medial wall decompression in the orbital apex, but this procedure is accompanied by a high risk of postoperative diplopia. In patients with no apical muscle crowding but with a presumptive orbital compartment syndrome the lateral technique should be considered with regard to the obviously missing influence on EOM motility [14].

Subjective pressure behind the globe as a sign of congestion is associated with a tight septum and a higher likelihood of DON. But it also becomes increasingly a more frequent indication for lateral wall decompression. Postoperatively there is dramatic resolution of the congestive component of this condition due to the release of orbital pressure (see the following).

Experimentally, the amount of measured orbital pressure release depends significantly on two factors: first, on removal of the orbital wall (virtually irrespective of the type of wall) and, second, on incision of the periorbita. Surprisingly, almost no increase in effect was achieved by adding further orbital walls [15].

## 4. The Role of Imaging Techniques

In conjunction with the typical clinical signs of TAO, ultrasonography is sufficient to diagnose the condition. If B-scans show enlarged muscle bellies with normal tendon size, the clinical diagnosis of TAO is confirmed. Internal muscle reflectivity in A- and B-scans may be inversely proportional to disease activity [16, 17]. 

Further information especially concerning the anatomical details and morphologic changes of the orbital soft tissues in the orbital apex can be assessed by computed tomography (CT) or magnetic resonance imaging (MRI). Magnetic resonance imaging (MRI) can be used to differentiate radiographically between active and inactive diseases. In TAO the extraocular muscles are isointense to normal muscle on T1-weighted MRI and hyperintense on T2 depending on the degree of oedema. The absence of oedema may demonstrate a fibrotic phase. The correlation of water content (oedema) and inflammatory activity can also be detected with MRI short-term inversion recovery (STIR) sequencing. Latest results on the predictive value of the signal intensity ratio (SIR) in MRI-TIRM suggest a correlation between SIR and the clinical activity score (CAS). To differentiate patients with active from inactive eyes disease a cut-off value of >2.5 at 1.5 Tesla was determined [18].

The disadvantage of MRI is the poor visualisation of bony structures, making it less suitable as a preparatory assessment for decompression surgery. In comparison CT displays an excellent view of the bony orbit and paranasal sinuses; an information that is mandatory if orbital decompression surgery is being considered.

## 5. History of Decompression Techniques

The earliest surgical approach in bony orbital decompression was published by Dollinger in 1911 [19]. He adapted Kroenlein's technique [20] for removal of an orbital dermoid cyst to decompress into the subtemporal fossa. The trans-frontal orbital roof decompression advocated by Naffziger in 1931 allowed access to both orbital apices in bilateral DON but again offered little in the way of proptosis reduction [21]. It is a time-consuming operation requiring the services of a neurosurgeon and is not without complications and risks. Moreover, the communication of the orbit and cranium predisposed to a pulsating exophthalmos which may persist [22].

Sewall's medial approach, introduced in 1936, involved the removal of the medial orbital wall by an external ethmoidectomy including, if necessary, the ethmoid cells and any air cells in the roof of the orbit as far back as the sphenoid sinus, thus allowing the orbital contents to expand medially towards the nose [23].

In 1950 Hirsch used a technique—first described by Lewkowitz in 1932 for orbital complications of paranasal sinusitis—which entailed removal of the orbital floor [24].

**Figure 1:**
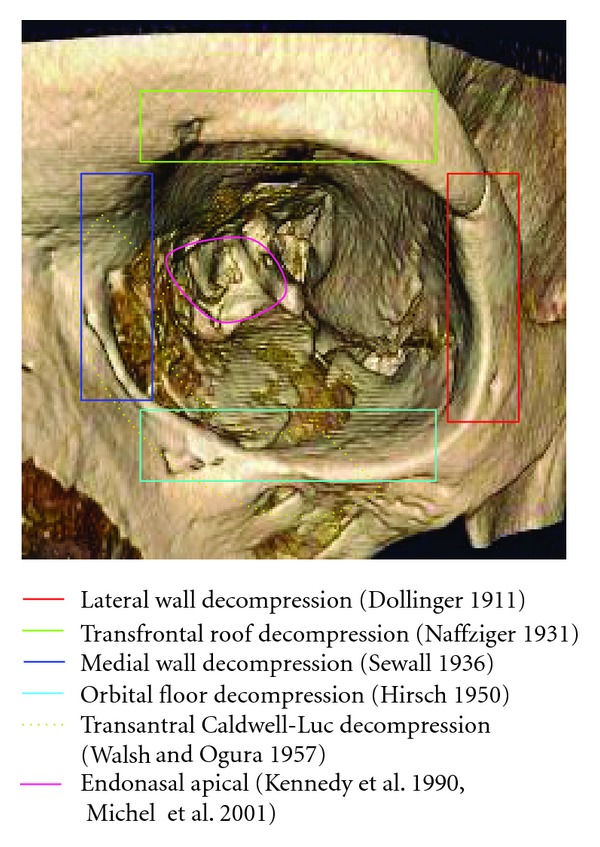
Different approaches to orbital decompression.

A combined approach described by Walsh and Ogura in 1957 involved a transantral Caldwell-Luc decompression of the medial and inferior orbital walls, which avoided external incisions [25]. This approach was widely accepted and used by many surgeons until the early 1980s. However, the high incidence of postoperative diplopia and infraorbital hypaesthesia and even pain were notable complications [26] that prompted the search for alternative approaches. An overview about the different approaches gives Figure 1.

The transnasal endoscopic approach addressing the medial orbital wall was first introduced by Kennedy et al. [27] in 1990 and by Michel et al. [28] in 2001. The efficacy of endonasal techniques has since been clearly demonstrated.

Over the ensuing decades numerous variations have been described, and these can be conveniently differentiated in terms of the approach employed—for example, transcaruncular [29, 30], transconjunctival [31], swinging eyelid [31, 32], or coronal incision [33–35]—or in terms of the number and amount of orbital walls removed [36, 37].

## 6. Techniques for Orbital Decompression Surgery

### 6.1. Bony Orbital Decompression (BOD)

Bony decompression may involve single or multiple walls of the orbit. Kikkawa et al. [38] have proposed a “graded orbital decompression based on the severity of proptosis.” Using the categories defined by Kalmann [37], these authors performed lateral orbital wall decompression with orbital fat removal if exophthalmos was less than 22 mm, additional medial wall decompression if exophthalmos was between 22 and 25 mm, and 3-wall decompression with removal of the orbital floor if exophthalmos was greater than 25 mm. 

The use of a coronal decompression has been detailed in various publications [33, 34, 37, 39, 40]. In most cases 3-wall decompression is attempted, which results in very effective exophthalmos reduction and improved aesthetic outcome. The main advantage is that the incision can be hidden in patients with an adequate hairline. Hidden incisions are certainly preferable, but they can also be camouflaged by using an upper eyelid crease incision or swinging-eyelid approach for the lateral wall, an inferior fornix transconjunctival incision for the orbital floor, and a transcaruncular incision or endonasal approach for the medial wall. There has been a trend in recent years to abandon the coronal approach in favour of the alternatives mentioned.

As mentioned before two-wall decompression involving the medial wall and the medial aspect of the floor was still the most popular approach until the 1980s. The high incidence of postoperative diplopia because of an inferior globe displacement was avoided by preserving the inferomedial strut located at the junction of the maxillary and ethmoid sinuses [31, 41].

“Balanced” decompression of the medial and lateral orbital walls has gained recent popularity because it may also lessen the occurrence of induced strabismus [35, 42]. It is postulated that this approach may limit inferomedial displacement of the globe and produce an equivalent prolapse of the medial and lateral rectus muscles into the newly created space [43]. 

In a retrospective study Goldberg et al. [42] demonstrated that balanced decompression is not more effective compared to deep lateral wall decompression alone in terms of average proptosis reduction (4.5 mm). Interestingly, preoperative strabismus resolved spontaneously in 25% of cases in the balanced decompression group and in 60% of cases in the lateral decompression group. New-onset strabismus was found in 33% in the balanced decompression group compared to just 7% in the lateral wall decompression group. Goldberg et al. [44] used CT to calculate the volume of bone available for removal in the deep lateral bony orbit. The “extended lateral orbit” was subdivided into three areas: the “lacrimal keyhole” (area around the lacrimal gland fossa), the “basin of the inferior orbital fissure” (the portion of the zygomatic bone and lateral maxilla and the area around the inferior orbital fissure), and the “sphenoid door jamb” (the thick trigone of the greater wing of the sphenoid which borders the inferior temporal fossa laterally and the middle cranial fossa posteriorly). The “sphenoid door jamb” makes the largest contribution to the total bone volume (5.6 mL) of the three areas potentially available for decompression. Proptosis reduction was as much as 6 mm. The authors estimated that 0.8 mm proptosis reduction might be achieved for every mL of bone removed [45]. 

In a recent publication Mehta and Durrani [46] presented their results after rim-sparing deep lateral wall decompression via canthal incision in 21 orbits where they found a comparable exophthalmos reduction of 4.8 mm with worsening of preexisting diplopia in 1 patient (6%).

An additional alternative for improving the effect on aesthetic rehabilitation is the insertion of subperiosteal orbital rim onlay implants, which are mostly used to camouflage remaining proptosis after decompression surgery [47]. Possible risks include lower eyelid restriction, implant infection, and visible implant edges.

The usefulness of endoscopic techniques for medial orbital decompression is still under evaluation. In an early, small series Kennedy et al. [27] reported improvement in visual acuity and globe protrusion in 9 out of 16 orbits. Lund et al. [48] showed mean improvements in axial proptosis of 4.4 mm with an endonasal approach compared to 3.8 mm with an external procedure.

Metson and Samaha [49] published an average exophthalmos reduction of 3.5 mm in a series of more than 100 patients. Worsening of strabismus after medial wall decompression is a well-known risk due to a shift of the muscle cone into the opened space of the ethmoid sinuses. Though Metson and Samaha [50] described the orbital sling technique to reduce the risk of motility disturbance following the endoscopic approach, medial wall decompression from our point of view should be reserved for patients with DON due to apical compression, or in the case of reconstructive surgery in patients with severe exophthalmos where maximal exophthalmos reduction is required. A prospective multicenter survey by the orbital surgeons of the EUGOGO group evaluated the outcomes of different techniques and approaches for orbital decompression for disfiguring exophthalmos being preferred around Europe [51]. They found exophthalmos reduction as a function of the number of orbital walls removed being increased by additional orbital fat resection. A significant improvement of quality of life was observed using the disease-specific quality of life questionnaire [52] with greatest improvement in the appearance score. As one might expect diplopia was the most common complication with a tendency of the swinging eyelid approach being beneficial compared to the other approaches. 

Whether stereotactic navigation in decompression surgery as described by Miller and Maloof [53] offers significant advantages remains to be proven.

### 6.2. Fat Removal Orbital Decompression (FROD)

As been described by Duke Elder [22] the futility of attempting to remove masses of orbital fat has been proved ever since the account published by von Graefe in 1864. Orbital fat excision may be performed alone or in combination with bony decompression, as mentioned above.

FROD for TAO was first described by Olivari in 1988 [54]. He reported “a significantly lower complication rate and higher success rate” compared with BOD after removal of 6 mL fat on average [55]. 

FROD as primary treatment for exophthalmos seems to be particularly well suited for patients who have a predominant volumetric increase in orbital fat. Careful imaging, preferably with MRI, is needed to discriminate between the tissue entities. 

Trokel et al. [56] performed fat excision from the superior nasal and inferior temporal orbital fat compartments. It should be noted that the average volume of fat is about 8 mL in a normal orbit but may be 10 mL or more in TAO patients. The authors demonstrated an average proptosis reduction of 1.8 mm with orbital fat excision alone, and the greatest average reduction in proptosis (3.3 mm) was produced in patients with preoperative Hertel measurements of greater than 25 mm. 

The original paper by Olivari [54] describes an average proptosis reduction of 6 mm resulting from an average removal of 6.2 mL fat. The author noted only a few complications, in particular a rate of new-onset strabismus of 4%. Unfortunately no details are provided concerning the methods of examination or the extent of change in motility. Adenis et al. [57] demonstrated that excision of a mean orbital fat volume of 7.3 ± 1.9 mL (range 3.25–12 mL) reduced proptosis on average by 4.7 ± 2.4 mm (range 1–11 mm). Reported side effects were few, being limited solely to ocular motility disturbances. Thereby main complications are temporary or even permanent motility problems, usually causing diplopia; in a later series Adenis et al. reported an incidence rate of new onset diplopia of 32% [58]. Nevertheless, assuming sound application of anatomical principles, orbital fat decompression can be a worthwhile method for achieving moderate exophthalmos reduction.

Sires et al. [59] performed an extensive study characterising human orbital fat and connective tissue, which were shown to contain many important structures in the intraconal and posterior trochlear fat regions. Their findings have implications for FROD because the inferior-lateral orbit was reported to be free of vital structures and therefore ideal for fat removal.

Our own experience with this technique discouraged us from continuing with the approach, particularly since the risk of induced motility disturbances after lateral wall decompression is negligible [14].

## 7. Our Approach to Bony Decompression

The following data result from a retrospective evaluation of the case record forms of 100 patients (75 women and 25 men) who had been decompressed in our clinic for TAO over a 7-year period from September 2003 to August 2010. 

### 7.1. Patients and Indications for Lateral Wall Decompression (LWD)

This section provides a retrospective review of a total of 100 patients (75 female, 25 male) with a mean age of 47.6 ± 12.7 years (range 20–74 years). They underwent lateral wall decompression (unilateral in 52 cases and bilateral in 48 cases, 148 orbits) performed by the same surgeon (RFG). The indications for surgery (multiple combinations possible) were as follows:

- exophthalmos/aesthetic rehabilitation (122 orbits);
- retrobulbar pressure (92 orbits);
- exposing keratopathy/lagophthalmos (13 orbits);
- DON with absence of MRI-proven apical muscle compression (compartment syndrome) (27 orbits).

### 7.2. Surgical Technique for LWD (Figure 2)

**Figure 2:**
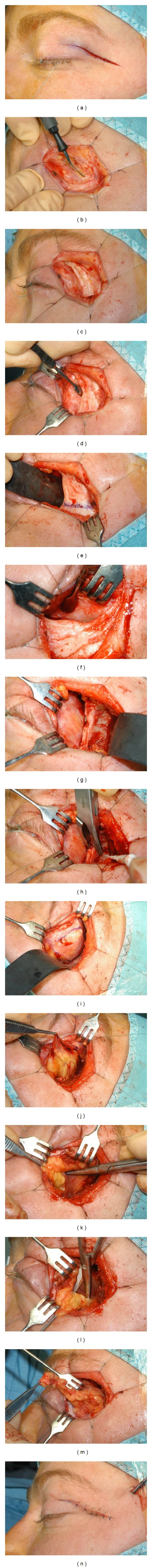
Surgical technique for LWD ((a)–(n)), see text for details).

Surgery is always performed under general anaesthesia. Surgery starts with a skin incision in the lateral third of the upper lid crease and follows a lateral sigmoid course over the zygomatic bone (Figure 2(a)). Blunt dissection performed by spreading the tips of Stevens scissors is continued to the lateral orbital rim. The lateral orbital wall is then dissected, entailing removal of the temporalis muscle until the periosteum is visible (Figure 2(b)). The periosteum is cut along the orbital rim and removed from the bone using a periosteal elevator (Figures 2(c) and 2(d)). From outside the temporalis muscle is blunt-dissected and can be held back with swabs, retractors, or traction sutures. The globe and the orbital soft-tissue contents are transferred nasally using malleable retractors (Figures 2(e) and 2(f)). Two osteotomies are performed next with an oscillating fine saw, one above the frontozygomatic suture, and the other at the beginning of the frontal process of zygoma (zygomatic arch). The lateral orbital wall is then removed (Figure 2(g)). The effect is increased if bone is additionally removed up to the height of the greater wing of the sphenoid (Figure 2(h)). The amount of removed bone fragment—which will not be replaced—is calculated. In our series the average volume was 1.6 mL.

The periorbit is opened (Figure 2(i)) and excised (Figure 2(j)), a procedure that is usually accompanied by a prolapse of orbital fat mainly from the inferolateral orbit (Figures 2(i) and 2(k)). Prolapsing fat can be carefully excised with minimal risk of orbital bleeding (Figures 2(k), 2(l), and 2(m)). Using this technique an average fat volume of 2.0 ± 1.0 mL was removed. Care should be taken to avoid removing fat from deep intraconal structures and by strong traction on fat pads. Besides the risk of orbital bleeding there is an additional risk of injuring or even destroying the small but important connective tissue ligaments—the rectus extraocular muscle pulleys [60]—which are necessary to allow normal movements of the globe. This has also been confirmed by Sires et al. in a postmortem study on exenteration specimens [59]. 

After insertion of a suction drainage system behind the globe, every layer is adapted and sutured separately (Figure 2(n)). To keep the globe axially retropositioned in the newly created space, a compression bandage is used for at least 24 hours. The amount of blood in the suction balloon and the pupillary light reaction are checked frequently within the first 12 hours postoperatively not to miss postoperative bleeding that could potentially lead to optic nerve compression and consecutive blindness.

### 7.3. Results of LWD

**Figure 3:**
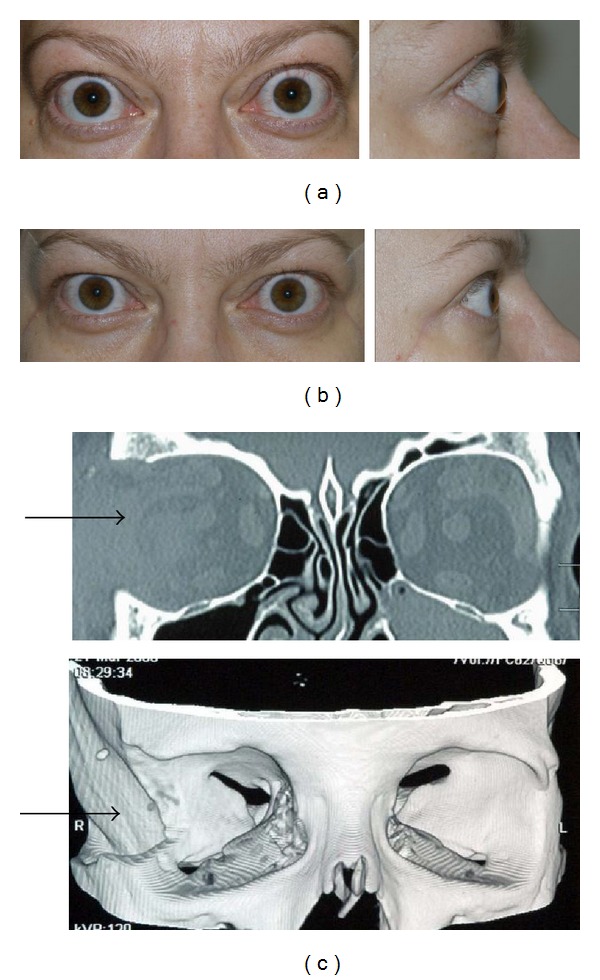
38-year-old female patient with significant exophthalmos and retrobulbar pressure sensation, no visual impairment. (a) Preoperatively: exophthalmos and lid retraction. (b) Postoperatively after lateral wall decompression: exophthalmos improved 5.0 mm on the right side and 4.0 mm on the left side. Additional lid surgery is needed (upper lid lengthening procedure). (c) Postoperatively: CT scan after lateral wall decompression of the right orbit (arrow: missing lateral orbital wall), coronal scan, and 3D reconstruction.

An example is given in Figure 3. 

The clinical outcome after lateral wall decompression in our patients was as follows.

**Reduction of Exophthalmos**

**Figure 4:**
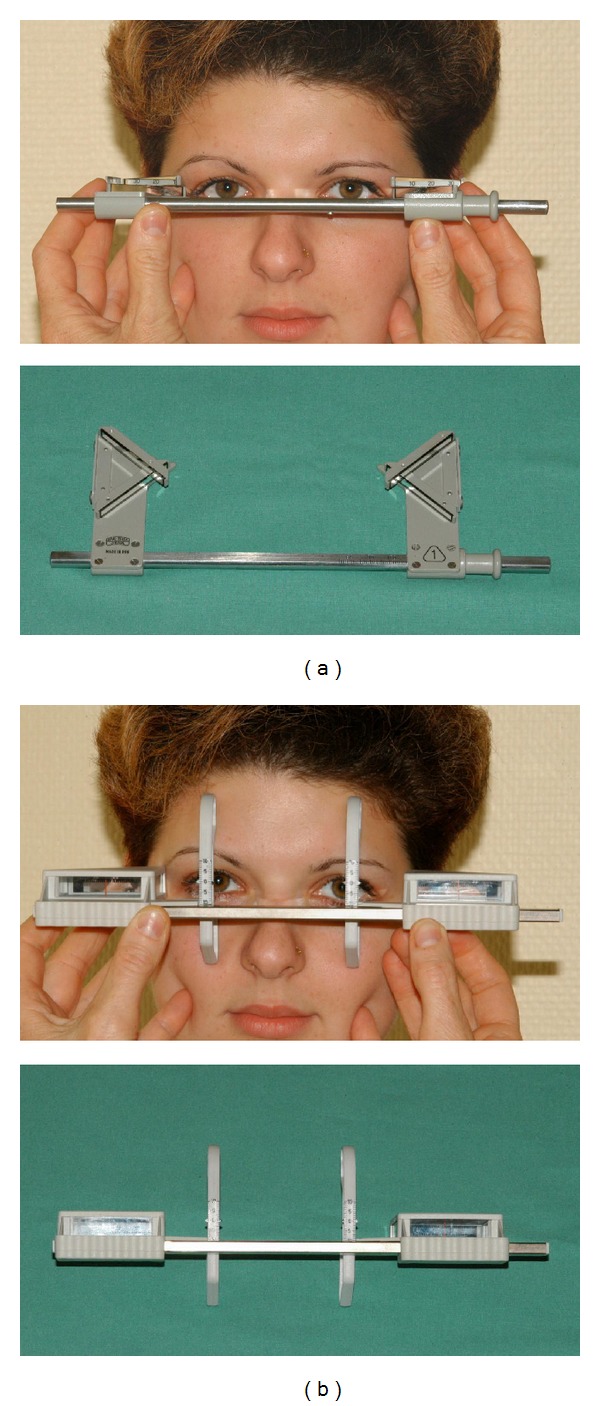
(a) Hertel exophthalmometer needs the lateral orbital rim as a reference point and is therefore not suitable after en bloc resection of the lateral orbital wall. (b) Naugle exophthalmometer uses the upper and lower orbital rims for fixation of the instrument.

Average exophthalmos reduction was 2.7 ± 1.4 mm with an average preoperative exophthalmos of 21.5 ± 2.5 mm (range14–30 mm) compared to 18.8 ± 2.2 mm (range 12–25 mm) postoperatively. It is important to note that lateral rim-based exophthalmometers (e.g., Hertel exophthalmometer) are not an appropriate tool following removal of the lateral orbital wall. Therefore we used the Naugle exophthalmometer pre- and postoperatively because the instrument uses the upper and lower orbital rim as a reference point (Figure 4 for comparison between Hertel/Naugle exophthalmometer).

**Retrobulbar Pressure Sensation**

Another important clinical feature prompting us to initiate lateral wall decompression is the often painful sensation of retrobulbar pressure. After surgery retrobulbar pressure sensation resolved completely in 88.5% and partially in 11.5% of patients in our population. Remarkably, the condition was not exacerbated in any of our patients as a result of the operation.

**Dysthyroid Optic Neuropathy (DON)**

Twenty patients (26 orbits) had optic neuropathy with an average visual acuity before decompression of 0.6 (range 0.05–1.0). Following lateral wall decompression only, visual acuity increased by an average of 0.3 in 11 of these orbits. Eleven orbits showed no increase in visual acuity. Two orbits even deteriorated after lateral wall decompression surgery; however, after additional apical endonasal decompression 1 week later, visual acuity improved to 0.7. Three orbits were lost to followup.

In those patients with no visual impairment before surgery but showing other signs of DON, for example, swelling of the optic nerve head, visual field defects, or pathological visual evoked potentials, these signs clearly improved after lateral decompression surgery.

**Disturbance of Motility**

In terms of the NOSPECS classification we noticed a preexisting restriction of motility in 105 out of 148 orbits (71%). After surgery 10 patients showed improvement of motility leading to improvement or resolution of preexisting diplopia in 4 of these patients. On the other hand eight patients showed slight worsening of motility with worsening of pre-existing diplopia in 2 patients and new onset diplopia in another 2 patients.

## 8. Possible Risks of Decompression Surgery

### 8.1. New Onset Diplopia and Postoperative Motility Disturbances

Nonconcomitant preoperative diplopia in patients with TAO may be due not only to inflammatory muscle changes or fatty degeneration and fibrosis of the muscle tissue itself but also to cicatricial adhesions at the surface of the muscle [61]. New-onset strabismus after orbital decompression may be secondary to muscle path changes that are induced by removal of an adjacent wall or by removal of periorbita, where some of the muscle pulleys insert [62]. 

Diplopia is a relatively common sequela of medial wall decompression and has a reported incidence of between 15% and 74% [28, 49]. In most cases this complication is thought to be the result of a change in the vector of pull of EOM. Motility disturbances that existed prior to medial wall decompression surgery do generally not improve or will even exacerbate.

Methods to reduce the incidence of new-onset postoperative diplopia after medial wall decompression include the preservation of a fascial sling of periorbit to prevent prolapse of the medial rectus muscle [63]. In DON patients with apical muscle crowding an alternative to a complete resection of the medial wall was described by Chu and coworkers [64]. They performed a selective decompression of the orbital apex in the deep orbit and spared the anteromedial and inferior orbital walls. They saw the advantage of this procedure in the reduced risk of postoperative diplopia though a sufficient decompression of the optic nerve was achieved.

**Figure 5:**
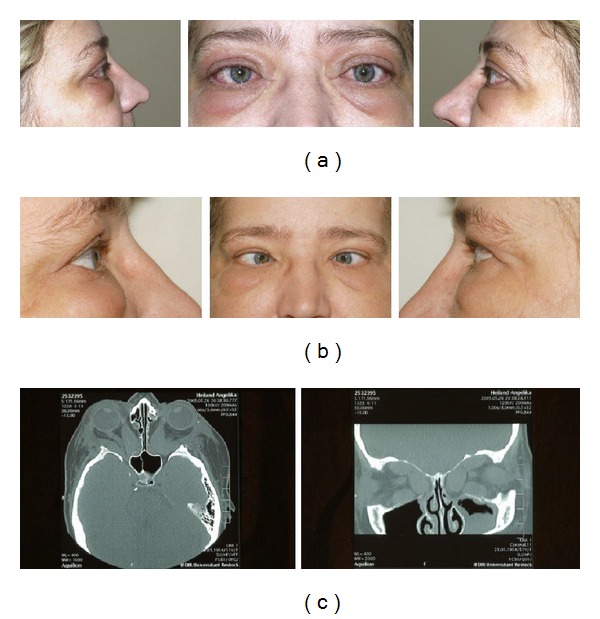
51-year-old female patient. (a) Preoperatively: severe active TAO with DON (visual acuity OD 0.6; OS 0.4), no diplopia. (b) Six months after combined medial and lateral wall decompression: visual acuity improved to OD 0.8 and OS 0.6; esotropia was induced leading to double vision in all directions of gaze. (c) Postoperatively: CT scan demonstrates definite movement of the rectus muscles into the newly created space in the paranasal sinuses and temporal fossa.

But from our experience patients with DON often require maximal decompression so that preservation of parts of the periorbit or even the bony orbit seems not favourable to us. Especially in these patients an extended excision of the periorbit is often required to allow the prolapse of soft tissue into the opened sinuses. In other words, where visual function is threatened, surgery should not be performed with respect to motility. It is important that patients are informed about the risk of worsening motility that leads to diplopia in most cases (example in Figure 5).

As has been stated in the above chapter on bony decompression techniques balanced decompression has been advocated to reduce the occurrence of induced strabismus [35, 42] because inferomedial displacement of the globe was prevented [43]. 

By comparing preoperative and postoperative CT scans, motility measurements, and Gorman score, we have demonstrated in our own series a clear displacement of the lateral rectus muscle after deep lateral wall removal [65] but without significant influence on motility (submitted) though we observed a worsening of motility in 4% (4 out of 100 patients) after lateral wall decompression with orbital fat resection. Comparable findings were described in a paper published by Goldbergs group [66]: even with their deep lateral decompression technique, no statistically significant effect was observed either on horizontal or on vertical motility.

This may explain why the concept of balancing-induced motility limitations on the medial and lateral rectus muscles is not completely satisfactory. It has long been known that the medial rectus muscle is more often affected by TAO than its lateral counterpart. Lateral decompression is usually performed much more frontally in relation to the equator of the belly of the adjacent lateral rectus muscle so that there is only minimal risk that the muscle cone in total will be displaced laterally, even in deep lateral decompression. In contrast in endonasal apical decompression, bone is removed in the region of the enlarged medial rectus belly, which consequently fills the newly created space in the neighbouring sinuses, including a shift of the muscle cone itself to the middle, thus amplifying or inducing esotropia as we have noted in so many patients. Further studies including cine-MRI investigations are needed to explain all the phenomena observed in the clinical setting [65].

Both the patient and the orbital surgeon should be aware that, in technical terms, strabismus surgery in TAO is sometimes difficult to perform but that diplopia in primary and reading position is usually curable [9, 67, 68]. Patients who have previously undergone decompression surgery have a lower overall primary success rate, have more muscles operated on, and therefore require a greater number of strabismus procedures [69]. Finally, nearly all patients may achieve a reasonable field of binocular single vision [9, 67]. This does not obviate the necessity to optimize decompression techniques to prevent or further reduce any effect on motility.

### 8.2. Postoperative Vision Loss

The most worrying complication of orbital decompression surgery is postoperative worsening or even loss of vision, which may occur intraoperatively, related to vascular or pressure damage to the optic nerve or globe, and postoperatively, related to orbital haemorrhage or vasospastic ischaemia. 

The literature contains only few information dealing with this particular problem. In a retrospective study of 1593 patients who had undergone an orbital surgical procedure, Bonavolontà [70] reported 7 cases of postoperative blindness (0.44%).

In the 2006 ESOPRS Mustardé Lecture, Rose reported a blindness rate of 0.56% following orbital surgery at the Moorfields Hospital between 1990 and 2005. The data were published in 2007 [71]. This study included 1350 orbital decompression procedures in TAO during the course of 2500 orbitotomies in the 15-year interval. Interestingly, all of the cases with postoperative vision loss occurred after surgical procedures other than orbital decompression, that is, incisional or excisional orbital tumor resections. 

Irrespectively, gentle surgical technique and good haemostasis during orbital decompression surgery are mandatory. We always use a suction drain to remove fluid from the operation site because we feel that this can reduce the risk of visual loss. Visual loss never occurred in our own patient series after orbital decompression surgery though one patient did experience vision loss due to intraorbital bleeding the day after removal of an orbital apex haemangioma. However, if loss of visual impairment is recognized postoperatively, rapid evaluation for evacuable haematoma or ischemic event is compelling. Patients should therefore remain in hospital until the drain has stopped evacuating blood or wound fluid and the drain has been removed. Although vision loss in orbital surgery is fortunately rare, it cannot be prevented entirely and must be kept in mind especially the first days after surgery. 

### 8.3. Other Risks

The risks specific to a coronal approach include facial nerve palsy, hair loss along the incision line—which might lead to scar visibility especially in man—and temporalis wasting. Possible complications of lateral wall decompression via the lid crease or swinging eyelid approach include temporary numbness in the distribution of the zygomatic or temporal-zygomatic trigeminal nerves. This symptom, which is often nontroublesome, may persist after surgery for up to one year. We observed one patient who reported chewing-related oscillopsia that was mild and not defacing. This phenomenon was also reported by others [1]. Temporalis wasting after disinsertion of the orbital rim—a complication feared by other authors and occasionally seen after coronal decompression [31, 39, 51]—is obviously caused by the disinsertion of the temporalis muscle from the lateral orbital wall. It was neither seen by Long and Ellis [72] who first described the en bloc resection of the lateral orbital wall nor by Leone and coworkers [73] who resected the lateral orbital wall together with the medial wall. In our own data we found one patient with a slight retraction around the scar area without significant hollowing of the temporalis region. As a consequence, care must be taken intraoperatively to ensure that any trauma to the temporalis muscle is only minimal with a precise layered adaption of the tissue planes. Dural laceration represents a rare complication during medial and lateral wall decompression irrespective of the approach. Remulla et al. [74] presented three cases of delayed postoperative infection more than 2 years after surgery for endoscopic orbital decompression. This was thought to be due to obstruction of the frontal sinus ostium by scar tissue or prolapsed orbital fat. The authors therefore recommended that the surgical technique be modified to leave the most anterosuperior portion of the lamina papyracea to prevent fat prolapse and scar formation into the region of the frontal recess.

## 9. Summary

A number of relatively safe surgical procedures for orbital decompression surgery currently exist, and the approach chosen will be governed by the experience available in the particular centre but should furthermore be tailored to the patient's needs. It is necessary to emphasize that proper decompression requires bracing or even removing the periorbit. The amount of proptosis reduction is influenced by preoperative Hertel values and is greater where exophthalmos is more severe.

Current trends in orbital decompression surgery account for the patients preoperative characteristics and intend to limit major complications. These include new-onset diplopia or worsening of preexisting motility deficits related to muscular fibrosis due to TAO and visible and disturbing scars which can be reduced or even avoided by camouflaging incisions (e.g., upper skin crease incision or swinging eyelid approach).

In the absence of DON we prefer the lateral wall decompression technique described above because of the following.

- The operation can be performed by the orbital surgeon himself/herself.
- Orbital anatomy can be readily visualized.
- The duration of surgery is not unreasonable.
- There is a low complication rate without major risks.
- In particular, there is no significant change in motility.

The indications for surgery have been influenced as the understanding and management of TAO have improved. There is also an increasing appreciation of the facial disfigurement caused by clinical signs, mainly by severe exophthalmos followed by lid retraction. As surgical techniques become more refined, surgeons are better prepared to address this problem. Because of the improved technique and relatively low risk, the lateral technique is also currently used for aesthetic rehabilitation.

In decompression for optic neuropathy, the key element is removal of the apical portion of the orbital walls, especially the medial wall. This is usually performed endoscopically in conjunction with an ENT surgeon. 

For the future a better understanding of the immunological and pathophysiological context of TAO should help to avoid severe and sight-threatening courses of the disease asking for aggressive surgical interventions in the active stage of the disease. But independently, the currently available surgical techniques overall represent save techniques to prevent blinding of the patient and furthermore to improve facial appearance and therefore to improve quality of life.
